# Carbon Nanofibers in Pure Form and in Calcium Alginate Composites Films: New Cost-Effective Antibacterial Biomaterials against the Life-Threatening Multidrug-Resistant *Staphylococcus epidermidis*

**DOI:** 10.3390/polym11030453

**Published:** 2019-03-10

**Authors:** Beatriz Salesa, Miguel Martí, Belén Frígols, Ángel Serrano-Aroca

**Affiliations:** Facultad de Veterinaria y Ciencias Experimentales, Universidad Católica de Valencia San Vicente Mártir, c/Guillem de Castro 94, 46001 Valencia, Spain; beatriz.salesa@ucv.es (B.S.); miguel.marti@ucv.es (M.M.); belen.frigols@ucv.es (B.F.)

**Keywords:** carbon nanofibers, calcium alginate, antibacterial activity, *Staphylococcus epidermidis*, multidrug-resistant bacteria, human keratinocyte HaCaT cells, cytotoxicity

## Abstract

Due to the current global health problem of antibiotic resistant recently announced by the World Health Organization, there is an urgent necessity of looking for new alternative antibacterial materials able to treat and impede multidrug-resistant infections which are cost-effective and non-toxic for human beings. In this regard, carbon nanofibers (CNFs) possess currently much lower cost than other carbon nanomaterials, such as graphene oxide, and exhibit excellent chemical, mechanical and electric properties. Furthermore, here, the first report on the antibacterial activity of CNFs was demonstrated. Thus, these nanomaterials, in pure form or incorporated in a minuscule amount into calcium alginate composite films to reduce production costs as much as possible, showed to be new weapons against a globally spreading multidrug-resistant pathogen, the methicillin-resistant *Staphylococcus epidermidis* (MRSE). This Gram-positive bacterium is becoming one of the most dangerous pathogens, due to its abundance on skin. In this study, these hollow filamentous materials, in direct contact with cells and loaded in the low-cost calcium alginate composite films, showed no cytotoxicity for human keratinocyte HaCaT cells, which render them very promising for biomedical applications. The CNFs used in this work were characterized by Raman spectroscopy and observed by high-resolution transmission electron with energy-disperse X-ray spectroscopy.

## 1. Introduction

Due to the capacity that bacteria possess of being able to develop antibiotic resistance by mutation, hospital associated infections, especially in immunocompromised and cancer patients, is increasingly spreading without suitable antibiotic therapy. The World Health Organization has recently shown the high levels of antibiotic resistance in both developed and undeveloped countries [1]. In fact, it has been estimated that deaths from microbial infections could surpass those from many other life-threatening diseases, such as cancer, by 2050 if no urgent actions are taken to address this serious situation. Therefore, there is an imperious necessity of looking for new alternative antibacterial materials and approaches capable of treating and preventing multidrug-resistant infections. 

Antibacterials usually include antibiotics, antimicrobial peptides (AMPs), metal ions/oxides, and quaternary ammonium compounds [2]. The utilization of antibiotics in an excessive or irresponsible manner have made them increasingly inefficient, due to the AR [3]. Effective antibacterials that have long been employed in a wide range of commercial products are metal ions and oxides [4]. However, many studies have shown that these kinds of materials can exhibit high toxicity for some types of mammalian cells [5]. Quaternary ammonium compounds can also induce drug resistance after long-term use [6]. Pure AMPs have currently high extraction cost despite their wide spectrum of antibacterial activities [7]. Thus, approaches to counter the production problems of AMPs have been conducted with peptoids [8], α-peptides [9] and β-peptides [10], but are still far from the pharmaceutical industry. In this regard, nanotechnology represents a promising emergent therapeutic strategy capable of facing the microbial resistance problem [11,12]. In addition, new nanotechnological approaches are being developed in order to produce new composite materials for biomedical applications as antibacterial weapons to combat multidrug-resistance bacteria [13], which could substitute traditional antibiotics [12,14]. Thus, their safe incorporation could bring a new revolution in the health sector [15]. 

Alternative materials, such as graphene and its derivatives, have shown intrinsic antibacterial activity and negligible cytotoxicity for human cells [16,17]. Thus, this type of nanomaterials represents an excellent alternative next-generation of antimicrobial agents. However, there are some studies that have stated that these carbon nanomaterials showed no antibacterial activity [18,19] and can be toxic for some human cells [20,21]. Therefore, it is still an open question which demands further investigation. 

Other carbon nanomaterials chemically very similar to graphene are carbon nanofibers (CNFs). These nanomaterials have shown to possess electric conductivity, which can be exploited to fabricate conductive composite biomaterials [22,23]. Besides, CNFs are currently manufactured at large scale, but at a much lower price (e.g., about 21 times lower price than graphene oxide (GO) when purchased from Sigma-Aldrich, Buchs, Switzerland). These carbon nanomaterials consist of hollow fibers mostly composed of carbon atoms [24] in the form of quasi one-dimensional filaments with excellent chemical, mechanical and electric properties [25]. Furthermore, in composite biomaterials, these filamentous materials can significantly enhance the water diffusion and mechanical properties of calcium alginate composite hydrogels with the addition of even a minuscule amount of them [26] like other nanomaterials, such as graphene oxide [27,28,29], which is very desirable for many potential bioengineering applications. 

Alginate is an important biopolymer approved by the US Food and Drug Administration (FDA) for human use in wound healing. It is water-soluble, composed of D-mannuronic and L-guluronic blocks, and can form hydrogels by crosslinking with calcium chloride. Thus, the polysaccharide hydrogel of calcium alginate is an excellent hydrophilic material for a wide range of industrial applications, such as water treatment [30], biodegradable packaging [31] and many biotechnological fields [32,33,34], because of its much lower cost than many other biopolymers and being nontoxic, biodegradable, biocompatible and renewable [35]. In the biomedical field, composites with CNFs have shown to be capable of promoting cardiomyocyte growth [36] and neural regeneration [37]. In the field of antibacterial materials, copper/zinc bimetal nanoparticles-dispersed CNFs [38] and porous CNFs containing antibacterial silver [39] have been recently developed as novel potential antimicrobial materials. However, the ability of bacteria to develop resistance mechanisms against Ag^+^ and Cu^2+^ as copper-resistant [40] and silver-resistant [41] mutant bacteria render these materials not very promising after long-term use. 

Therefore, in the present study, even though the controversial results are shown in literature for other carbon nanomaterials, we hypothesized that CNFs in pure form and incorporated in calcium alginate composite films could also possess antibacterial activity and no cytotoxicity for human cells. Due to the importance of finding new alternative antibacterial agents against multidrug-resistant pathogens, the antibacterial activity of these materials was tested against a multidrug-resistant pathogen, the life-threatening methicillin-resistant *Staphylococcus epidermidis* (MRSE), which is a leading cause of catheter-associated disease, particularly among low birth weight premature infants [42]. Furthermore, it has been recently reported that there is imperious need of finding new antibacterial materials able to kill this pathogen, because it is globally spreading and has evolved to become a formidable nosocomial pathogen [43]. Since *S. epidermis* is a conspicuous member of the human microbiome, widely present on healthy skin [44], the cytotoxicity of these materials was tested in the presence of human keratinocyte HaCaT cells in order to study their potential biomedical applications. 

## 2. Materials and Methods 

### 2.1. Materials 

Calcium chloride (≥93.0%) and zinc chloride (≥97.0%) from Sigma-Aldrich (Buchs, Switzerland), carbon nanofibers (CNFs, Graphenano, Yecla, Spain) and sodium alginate (Panreac AppliChem, Darmstadt, Germany) were used as received. Tryptic soy broth (TSB) and tryptic soy agar (TSA) from Liofilchem (Teramo, Italy), and the Gram-positive multidrug-resistant MRSE, RP62A [45], was used in the antibacterial tests. Non-tumorigenic immortalized human keratinocyte HaCaT cells were supplied by the Medical Research Institute Hospital La Fe, Spain. DMEM medium containing 10% FBS (Biowest SAS, Nuaillé, France), penicillin (Lonza, Verviers, Belgium) and streptomycin (HyClone, GE Healthcare Life Sciences, Logan, UT, USA), dimethyl sulfoxide (DMSO, Sigma Aldrich, Buchs, Switzerland) were used in the cytotoxicity tests. Isotonic saline solution (NaCl 0.9% *w/v*) from FisioVet (B. Braun VetCare SA, Barcelona, Spain) was used in the antibacterial and cytotoxicity tests of the CNFs in pure form. 

### 2.2. Synthesis of Calcium Alginate/CNFs Composite Films

Calcium alginate with a very low amount of CNFs (0.1% *w/w*) was cross-linked with calcium chloride. Thus, 0.00025 g of CNFs was dispersed in 22 mL of distilled water. After that, 0.25 g of sodium alginate was dissolved in this CNFs/water dispersion with a magnetic stirrer for 1 h at 24 ± 0.5 °C. This mixture was poured into a Petri dish and left for 24 h in an oven at 37 °C to form thin films by solvent evaporation. Finally, the produced films were soaked in 2% *w/v* aqueous CaCl_2_ solution for 2 h. Thus, after rinsing with distilled water for three times, the films were vacuum dried at 60 °C ± 0.5 °C to constant weight. Control calcium alginate films were also prepared following the same procedure without the initial addition of CNFs. 

The sodium alginate utilised to produce the calcium alginate/CNFs films was characterized by the NOBIPOL group at the Norwegian University of Science and Technology. Thus, size exclusion chromatography with multi angel light scattering (SEC-MALS) detection was performed for molar mass and mass distribution determination. High performance anion-exchange chromatography with pulsed amperiometric detection (HPAEC-PAD) was employed to determine the block length distribution of the alginate chains. Finally, the sodium alginate was analysed by nuclear magnetic resonance (NMR) spectroscopy to obtain the fraction of M and G blocks (mono, di and triades). 

### 2.3. Antibacterial Tests

#### 2.3.1. Antibacterial Test for CNFs 

A previous study of the antibacterial properties of another carbon nanomaterial, graphene oxide, has shown strong antibacterial activity against the bacterial model *E. coli* for a concentration of 80 µg/mL in isotonic saline solution (to avoid interaction between medium compounds and GO) and 2 h of direct contact [46]. Therefore, following the same procedure described in that study, the antibacterial activity of CNFs were tested by direct contact with MRSE cells using the same concentration and time exposure. Thus, a sterile dispersion of CNFs in isotonic saline solution at 80 µg/mL, and autoclaved at 121 °C for 15 min, was prepared by sonication for 2 h right before being utilized in the bacterial cultures. The bacteria were grown in TSB medium at 37 °C into a shaking incubator at 140 rpm overnight. A dilution of this culture was performed and harvested in the mid-exponential growth phase until obtaining cell samples containing 10^6^ to 10^7^ colony forming units per mL (CFU/mL). A UV/VIS Nanocolor UV0245 spectrophotometer (Macherey-Nagel, Düren, Germany) was used at 540 nm to determine these bacterial concentrations. Cultures were centrifuged at 600 rpm (3952× *g*) for 10 min to pellet cells. Then, the cells were washed three times with isotonic saline solution in order to remove any type of residual medium component. The bacteria were then resuspended in fresh sterile CNFs’ dispersion to be incubated at 37 °C under 250 rpm shaking speed for 2 h. This high shaking speed was selected in order to reduce precipitation of carbon nanofibers during the culture time as performed in reference [46]. The loss of viability of the microorganisms was analysed by the colony counting method. A volume of 100 μL of each cell dilution was spread onto TSA plates, and left to grow overnight at 37 °C. Thus, loss of viability (*LV*) can be calculated from the colony counting results as the decrease of CFU/mL from the initial concentration at time 0 (*C*_0_) to that achieved at 2 h of culture (*C*) divided by the initial concentration (*C*_0_) as expressed in Equation (1) [46].

(1) $$LV\left(\%\right)=\frac{C_{0}-C}{C_{0}}\cdot 100$$

The microorganism cultures were placed in 15 mL tubes located in a Certomat IS orbital shaking incubator (Sartorius Stedim Biotech, Germany) at 37 °C for 2 h. This antibacterial test was performed four times on different days to provide reproducible results. A well-known antibacterial agent (zinc [47]) with a concentration of 80 µL/mL in isotonic saline solution was prepared with zinc chloride to be used as positive antibacterial control. 

#### 2.3.2. Antibacterial Test for Calcium Alginate/CNFs Composite Film

The antibacterial activity of the composite films of calcium alginate/CNFs was tested by the agar disk diffusion test [2,48]. Before starting these antibacterial assays, each disk was sterilized with ethanol and ultraviolet (UV) radiation for 1 h (per each side) in a laminar flow hood with a 12.0 W lamp of UV-C radiation. Lawns of MRSE, resuspended on TSB, of about 1.5 × 10^8^ CFU/mL were cultivated on TSA plates. The sterilized sample and control disks were carefully placed upon the lawns of bacteria to be incubated aerobically at 37 °C for 24 h. The antibacterial action of the tested materials on the growth of the MRSE bacteria was expressed as normalised width of the antimicrobial “halo” (*nw_halo_*), calculated with the inhibition zone diameter (*d_iz_*) and sample disk diameter (*d*) in Equation (2) [2]. These diameter values were determined with the Image J analysis software.

(2) $$nw_{halo}=\frac{\frac{d_{iz}-d}{2}}{d}$$

Each antibacterial test was performed in quadruplicate in four different days to ensure reproducible results. Thus, the mean and standard deviation of the normalised antibacterial “halos” were determined. 

### 2.4. Cytotoxicity Assays 

Human keratinocyte HaCaT cells were cultured at 37 °C in DMEM containing 10% Fetal bovine serum (FBS), 100 units/mL penicillin and 100 mg/mL streptomycin in a 5% CO_2_ incubator. These experiments were performed four times in order to obtained representative results.

#### 2.4.1. Cytotoxicity Assay for CNFs 

The cell viability of the human keratinocyte HaCaT cells in the presence of CNFs was determined by the methylthiazolyldiphenyl-tetrazolium bromide (MTT) assay. Cells were cultured into a 96-well plate at a density of 5 × 10^5^ cells/well. After 24 h of incubation, the culture medium of each well was replaced with 100 µL of CNFs dispersed in isotonic saline solution at 80 µg/mL, which is the same concentration that was used in the antibacterial tests. This dispersion of CNFs was prepared by sonication during 2 h and used immediately. A positive control of 100 µL of isotonic saline solution without CNFs was also used to replace the culture medium. After 3 h of incubation (higher time exposure than in the antibacterial test), the cells were incubated with 5 mg/mL MTT in each well for 4 h. The formazan was solved in 100 µL dimethyl sulfoxide at 24 ± 1 °C. Finally, absorbance readings were carried out on a Varioskan microplate reader (Thermo Fisher Scientific, Barcelona, Spain) at 550 nm. 

#### 2.4.2. Cytotoxicity Assay for Calcium Alginate/CNFs Composite Film Extracts

Specimens of calcium alginate/CNFs and calcium alginate (control) films with an approximate diameter of 10 mm were cut with a cylindrical punch. The samples were sterilized under ultraviolet light for 1 h per each side. The disks (n = 4) were placed into a 12-well plate with 1 mL of DMEM without FBS per well. The ISO-10993 standard recommendations were followed in these assays. Thus, 3 cm^2^/mL was used according to this norm. After incubation in humidified CO_2_/air (5/95%) atmosphere at 37 °C for 72 h, the extracts were collected and filtered (0.20 µm) for immediate use in the cytotoxic tests. Cells were cultured into a 96-well plate at 5 × 10^5^ cells/well density. After 24 h of incubation in a 5% CO_2_ humidified atmosphere at 37 °C, the medium present in each well was replaced with 100 µL of the calcium alginate/CNFs composite film extract, the calcium alginate film extract, 100 µL of negative control (same medium used to produce the film extracts) and 100 µL of positive control, which consisted of 100 µL of 1000 µM zinc solution prepared with zinc chloride. This zinc concentration is over the cytotoxicity level (100 µM) for mesenchymal stromal cells [49]. Cell incubation in each well was performed with 5 mg/mL MTT for 4 h. The formazan crystals were solved in 100 µL of DMSO at 24 ± 1 °C. Finally, absorbance readings were performed on the microplate reader at 550 nm.

### 2.5. Electron Microscopy

The morphology of the CNFs were analysed by high-resolution transmission electron microscopy (HR-TEM) with a JEM 2100F (JEOL, Tokyo, Japan) 200 kV electron microscope. This HR-TEM microscope is equipped with energy-disperse X-ray spectroscopy (EDS) for the elemental analysis at 20 kV of voltage. The CNFs were dispersed in dichloromethane in an ultrasound bath for ten minutes and subsequent drying at ambient temperature was performed before HR-TEM observation.

The morphology of the calcium alginate/CNFs composite films was observed with a JEM-1010 (JEOL, Japan) 100 kV transmission electron microscope (TEM). TEM sample preparation consisted of producing ultrathin samples with sections of 60 nm. A Leica Ultracut UC6 ultramicrotome (Leica Mikrosysteme GmbH, Wien, Austria), a Diatome diamond knife (Diatome Ltd., Bienne, Switzerland) were used for the ultrathin cutting. TEM grids (300 mesh) coated in carbon film were utilised to place the TEM specimens for observation. 

### 2.6. Raman Spectroscopy

Raman spectroscopy was performed onto a glass disk with a Renishaw inVia confocal micro-Raman spectrometer with an argon ion laser at 633 nm from 1000 to 3000 cm^−1^ with ×20 lens at 600 L·mm^−1^ grating. 

### 2.7. Statistical Analysis 

The results obtained in this study were analysed by ANOVA. Subsequent multiple comparisons were conducted using the Tukey’s post-hoc analysis at a significant level of at least *p* < 0.05. 

## 3. Results and Discussion

### 3.1. Morphology and Raman spectroscopy

**Figure 1:**
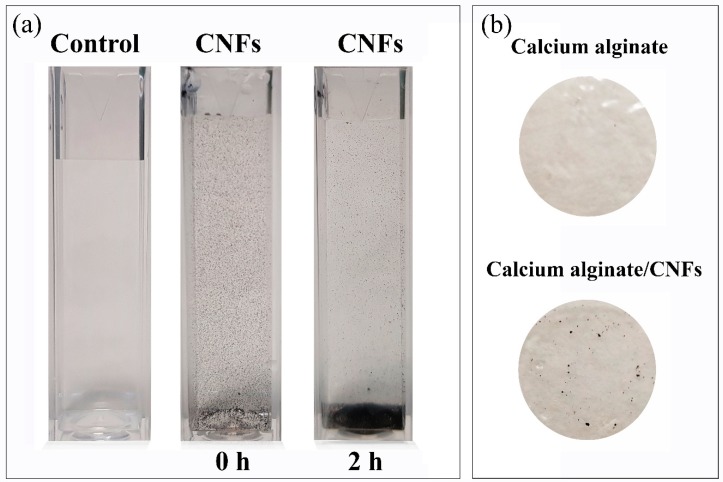
Dispersion of carbon nanofibers at 80 µg/mL after sonication (0 h) and after the dispersion was idle for 2 h (**a**), and films of calcium alginate and calcium alginate/CNFs (carbon nanofibers) (**b**).

Dispersion of carbon nanofibers and calcium alginate/CNFs composite films were prepared as described in the materials and methods section. Photographs of the CNFs’ dispersion (at 80 µg/mL) and the alginate-based films are shown in Figure 1. It is important to notice how most of the carbon nanofibers precipitate after the dispersion was idle for 2 h, due to the hydrophobic and non-polar nature of these carbon nanomaterials. Thus, the distribution of the hydrophobic CNFs in the hydrophilic polymer matrix of alginate becomes quite heterogeneous, due to this strong difference of polarity (see Figure 1b). 

**Figure 2:**
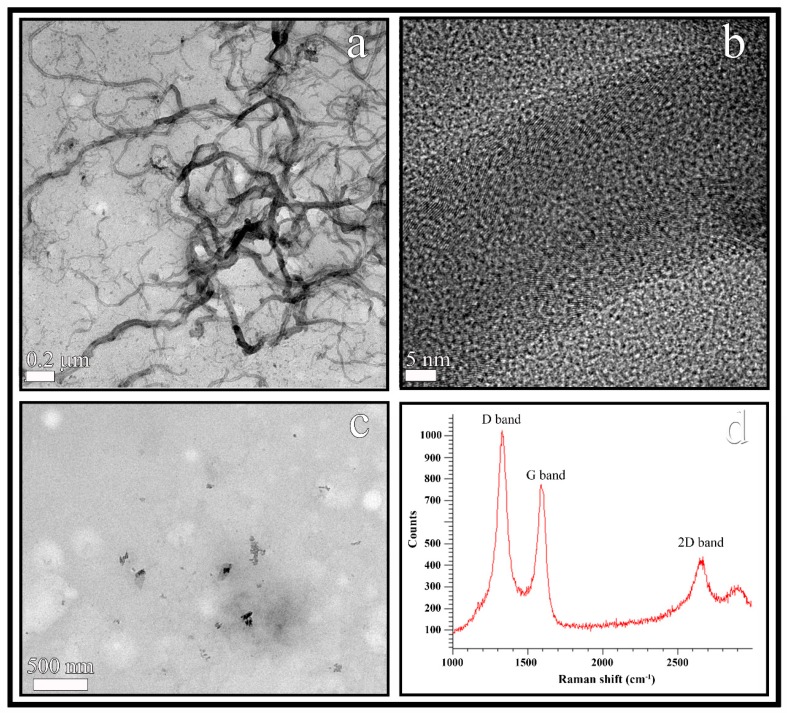
HR-TEM images of the carbon nanofibers at two different magnifications (**a**,**b**), TEM of cost-effective calcium alginate/CNFs composite films (**c**) and Raman spectrum of carbon nanofibers (**d**).

The CNFs were observed by HR-TEM and showed a morphology of hollow fibers with a wide range of diameters varying from 10 to 100 nm and irregular lengths ranging from a few nm to some µm (see Figure 2a). The black spots appearing in the HR-TEM micrograph of the CNFs at higher magnification (Figure 2b) were analysed to confirm the van der Waals radius of carbon [50], that is, a distance of 3.42 Å. In addition, the EDS analysis revealed that these carbon nanofibers are composed of 95.8% *w/w* of carbon atoms and a low percentage of oxygen atoms (4.2% *w/w*). 

Calcium alginate/CNFs composite films were prepared with a minuscule amount (0.1% *w/w*) of CNFs in order to produce cost-effective composite materials. Thus, the TEM micromorphology of these composite films is shown in Figure 2c. The CNFs (dark phase) are embedded and randomly distributed in the hydrophilic alginate biopolymer matrix (clear phase) as expected, due to these carbon nanomaterials are hydrophobic and non-polar in nature [51].

Raman spectroscopy is usually utilized to study structural defects and ordered/disordered of carbon nanotubes and nanofibers [52]. Thus, the Raman spectroscopy of the CNFs utilized in this study showed a strong D band and a broad G band at approximately 1350 and 1580 cm^−1^ respectively as expected [26,52] (see Figure 2d). Structural defects, edge effects and dangling sp^2^ carbon bonds breaking symmetry are related to the appearance of the D Raman band in carbon nanomaterials. The D band/G band intensity ratio (I_D_/I_G_) is usually employed as a measure of the defect/disordered carbon structure [53]. In this analysis, the I_D_/I_G_ ratio showed a value close to 1.5 confirming a significant degree of disorder typical of irregular carbon structures [52]. Moreover, the high 2D band at approximately 2690 cm^−1^ also indicates a high degree of disorder [52] supporting the chemical nature of the CNFs.

### 3.2. Antibacterial Tests

**Figure 3:**
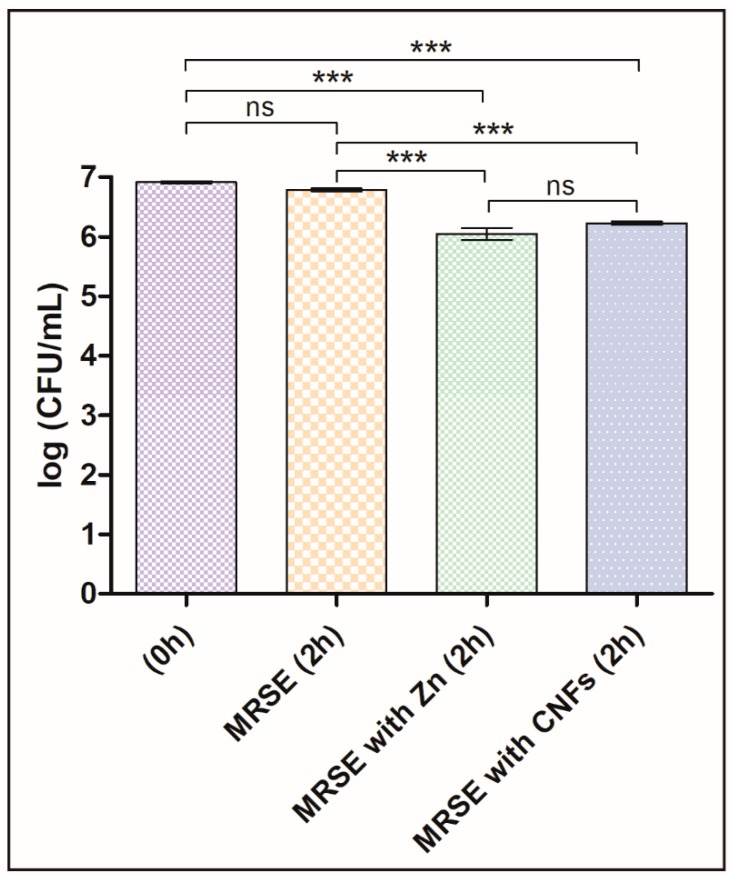
Multidrug-resistant *Staphylococcus epidermidis* (MRSE) cultured in isotonic saline solution, in isotonic saline solution with 80 µg/mL of zinc (MRSE with Zn) and in isotonic saline solution with 80 µg/mL of carbon nanofibers (MRSE with CNFs) at time zero and after 2 h of culture at 37 °C. Error bars represent standard deviation. The one-way ANOVA results are indicated in this figure. * *p* < 0.05, ** *p* < 0.001 and *** *p* < 0.0001 and ns indicates “no significant difference” (*p* > 0.05).

The antibacterial properties of CNFs were studied here for the first time in literature. Thus, we present the first evidence of CNFs as new antibacterials able to kill a multidrug-resistant pathogen by direct contact of CNFs with MRSE cells for two hours. The results are compared with a control culture without CNFs (negative control) and a positive control with a well-known antibacterial agent, zinc, in Figure 3. The results of these tests showed that the statistically significant differences between log-transformed CFU/mL at different times with and without CNFs confirmed our hypotheses that CNFs possess antibacterial activity as successfully achieved with other carbon nanomaterials at this concentration and time exposure [46]. Furthermore, the antibacterial activity of CNFs was not statistically different from that achieved with a positive control. A slight decrease of the CFU/mL of the negative control sample was expected, because they were grown in isotonic saline solution without growth medium for 2 h. However, this decrease in the log-transformed CFU/mL was not statistically significant. 

**Figure 4:**
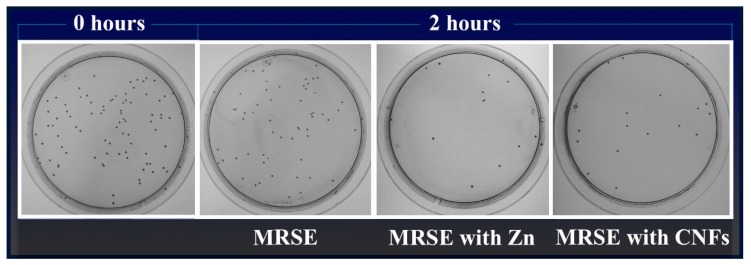
Representative plate images with the Colony Forming Units (CFU) obtained in the antibacterial test of multidrug-resistant *Staphylococcus epidermidis* (MRSE) cultured in isotonic saline solution, in isotonic saline solution with 80 µg/mL of zinc (MRSE with Zn) and in isotonic saline solution with 80 µg/mL of carbon nanofibers (MRSE with CNFs) at time zero and after 2 h of culture at 37 °C (Dilution factor of 10^−4^).

Figure 4 illustrates representative plate images of the antibacterial results for carbon nanofibers dispersed in isotonic saline solution at 80 µg/mL in direct contact with MRSE cells at time zero and after 2 h of culture at 37 °C. Representative plate images of the negative (MRSE) and positive (MRSE with Zn) controls are also shown.

As already mentioned, the difference of cell death of MRSE cultivated in isotonic saline solution in the presence of CNFs or in the presence of zinc was not statistically significant (see Figure 3). Thus, the loss of viability, calculated as the decrease of CFU/mL from time 0 to 2 h of culture at 37 °C by Equation (1), was 79.4 ± 2.4% for MRSE with CNFs and 85.9 ± 5.0% for MRSE with Zn. These results are in good agreement with that previously reported for the GO carbon nanomaterial against Gram-negative *E. coli* for the same concentration and time exposure [46]. 

In order to confirm the antibacterial activity of CNFs and their potential use for the development of new antimicrobial nanocomposites, another antibacterial test was performed by the agar disk diffusion test [2,48]. Thus, the antibacterial activity of the sterilized films of calcium alginate/CNFs and calcium alginate without CNFs as control sample, in the form of 10 mm disks, were tested on MRSE. The characterization of the sodium alginate used in the synthesis of alginate-based films showed a guluronic acid content of 43% distributed in blocks of dimers (27%) and trimmers (23%). This analysis showed also a weight-average molecular weight (M_w_) of 379.5 ± 9.5 KDa and number-average molecular weight (M_n_) of 170.7 ± 3.1 KDa.

**Figure 5:**
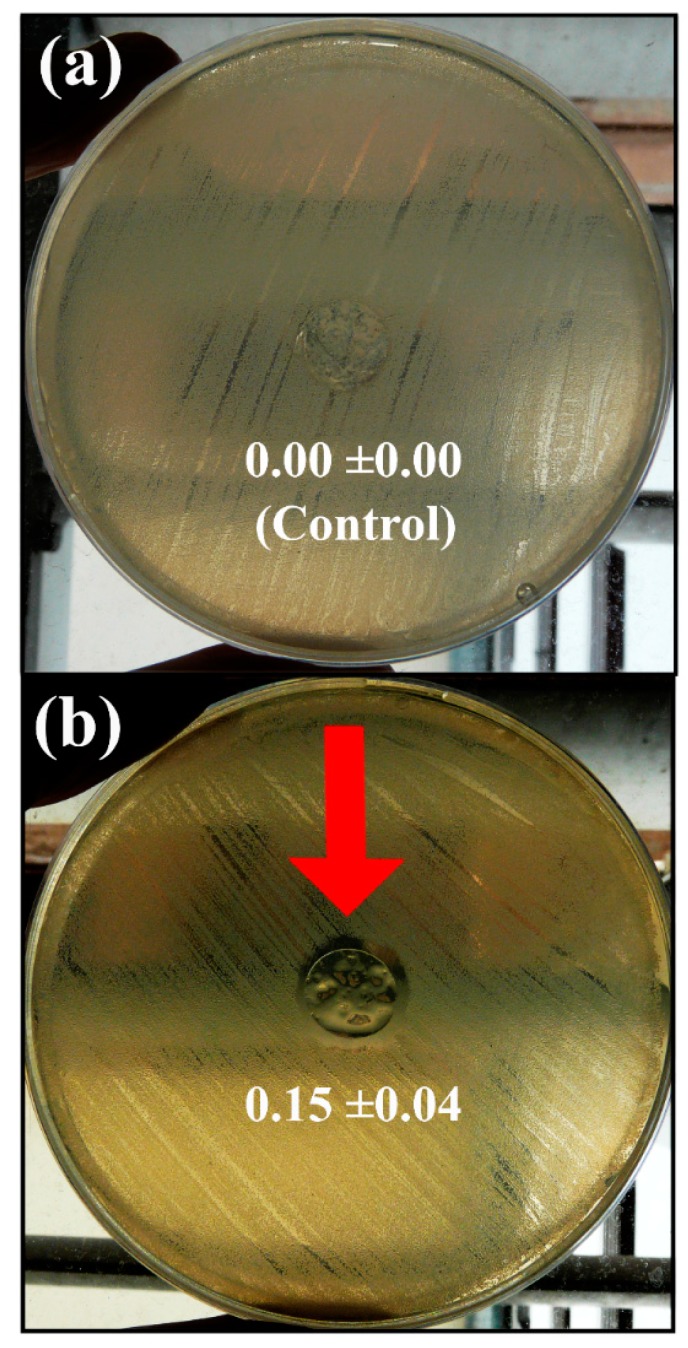
Antibacterial activity of the calcium alginate films without carbon nanofibers (control sample disk) (**a**) and calcium alginate/carbon nanofibers composite films (**b**) against multidrug-resistant *Staphylococcus epidermidis* by the agar disk diffusion test after 24 h of culture at 37 °C. The red arrow indicates the sample disk showing antibacterial “halo” and normalised widths (*nw_halo_*) calculated with Equation (2) are shown for each image.

The results of the agar disk diffusion test showed that the calcium alginate/CNFs composite films possess antibacterial activity against MRSE forming a significant inhibition zone around the sample disk in comparison with the calcium alginate films without CNFs (control disk) after 24 h of culture at 37 °C (see Figure 5a,b). 

Therefore, these results confirm that CNFs in pure form or incorporated into low-cost calcium alginate composite films can be exploited as new nanotechnological approaches against the MRSE multidrug-resistant-bacteria. Even though the antibacterial action mechanism of carbon nanomaterials, such as graphene oxide is still an open question, it is often associated with membrane disruption, bacteria wrapping, electron transfer mechanism and induction of oxidative stress by reactive oxygen species (ROS) [54,55]. Here, the observed antibacterial activity of CNFs by direct contact with MRSE cells and by the agar disk diffusion test shows somehow that several of these mechanisms might be involved.

### 3.3. Cytotoxicity Tests

**Figure 6:**
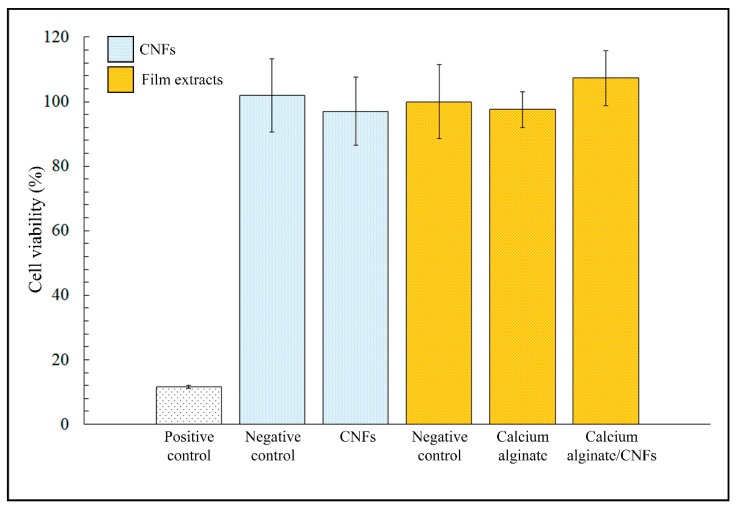
MTT cytotoxicity results of CNFs dispersed in isotonic saline solutions, calcium alginate film extracts, calcium alginate/CNFs composite film extracts, positive and negative controls cultured in the presence of human keratinocyte HaCaT cells at 37 °C. Differences in cell viability between negative controls and samples were no statistically significant (*p* < 0.05).

Figure 6 shows the MTT cytotoxicity results of CNFs dispersed in isotonic saline solutions, calcium alginate film extracts, calcium alginate/CNFs composite film extracts in the presence of human keratinocyte HaCaT cells cultured at 37 °C. 

No statistically significant differences in cell viability were found between the negative controls and samples of dispersed CNFs. Therefore, these results showed that CNFs are non-toxic for human keratinocyte HaCaT cells (~100% viability) at the same concentration and higher time exposure than those used in the antibacterial tests. Furthermore, the calcium alginate/CNFs composite film extracts obtained after incubation in DMEM during 72 h did not show any statistically significant cytotoxic effect either for this low amount of carbon nanomaterials (0.1% *w/w*). 

## 4. Conclusions

We have demonstrated the antibacterial properties of CNFs for the first time. Furthermore, these carbon nanomaterials in pure form or incorporated in a minuscule amount to produce low-cost calcium alginate composites were able to kill the important multidrug-resistant *Staphylococcus epidermidis*, which is spreading very fast in hospitals and is considered currently one of the most potential pathogens. The cytotoxic assays showed that CNFs are non-toxic for human keratinocyte HaCaT cells at the same concentration and higher time exposure than those used in the antibacterial tests. Furthermore, the MTT tests of the calcium alginate/CNFs composite film extracts did not show any cytotoxic result in the presence of this type of cells. Therefore, these carbon nanomaterials in pure form or in calcium alginate composite films constitute a very promising cost-effective approach for the treatment and prevention of MRSE multidrug-resistant infections in biomedical applications.
